# Living on the edge: environmental variability of a shallow late Holocene cold-water coral mound

**DOI:** 10.1007/s00338-022-02249-4

**Published:** 2022-04-12

**Authors:** Jacek Raddatz, Volker Liebetrau, Andres Rüggeberg, Anneleen Foubert, Sascha Flögel, Dirk Nürnberg, Karen Hissmann, Johannes Musiol, Tyler Jay Goepfert, Anton Eisenhauer, Wolf-Christian Dullo

**Affiliations:** 1 Institute of Geosciences, Goethe University Frankfurt, Altenhöferallee 1, 60438 Frankfurt, Germany; 2 GEOMAR Helmholtz Centre for Ocean Research Kiel, Wischhofstr. 1-3, 24148 Kiel, Germany; 3 Department of Geosciences, University of Fribourg, Chemin du Musée 6, 1700 Fribourg, Switzerland; 4 Present Address: School of Earth & Space Exploration, Arizona State University, Tempe, AZ 85287-1404 USA

**Keywords:** *Desmophyllum pertusum*, Deep-sea ecosystems, Mg/Ca, *Lobatula lobatula*, Computed tomography, Th/U dating, Seawater density, Oxygen isotopes

## Abstract

Similar to their tropical counterparts, cold-water corals (CWCs) are able to build large three-dimensional reef structures. These unique ecosystems are at risk due to ongoing climate change. In particular, ocean warming, ocean acidification and changes in the hydrological cycle may jeopardize the existence of CWCs. In order to predict how CWCs and their reefs or mounds will develop in the near future one important strategy is to study past fossil CWC mounds and especially shallow CWC ecosystems as they experience a greater environmental variability compared to other deep-water CWC ecosystems. We present results from a CWC mound off southern Norway. A sediment core drilled from this relatively shallow (~ 100 m) CWC mound exposes in full detail hydrographical changes during the late Holocene, which were crucial for mound build-up. We applied computed tomography, ^230^Th/U dating, and foraminiferal geochemical proxy reconstructions of bottom-water-temperature (Mg/Ca-based BWT), δ^18^O for seawater density, and the combination of both to infer salinity changes. Our results demonstrate that the CWC mound formed in the late Holocene between 4 kiloannum (ka) and 1.5 ka with an average aggradation rate of 104 cm/kiloyears (kyr), which is significantly lower than other Holocene Norwegian mounds. The reconstructed BWT_Mg/Ca_ and seawater density exhibit large variations throughout the entire period of mound formation, but are strikingly similar to modern in situ observations in the nearby Tisler Reef. We argue that BWT does not exert a primary control on CWC mound formation. Instead, strong salinity and seawater density variation throughout the entire mound sequence appears to be controlled by the interplay between the Atlantic Water (AW) inflow and the overlying, outflowing Baltic-Sea water. CWC growth and mound formation in the NE Skagerrak was supported by strong current flow, oxygen replenishment, the presence of a strong boundary layer and larval dispersal through the AW, but possibly inhibited by the influence of fresh Baltic Water during the late Holocene. Our study therefore highlights that modern shallow Norwegian CWC reefs may be particularly endangered due to changes in water-column stratification associated with increasing net precipitation caused by climate change.

**Supplementary Information:**

The online version contains supplementary material available at 10.1007/s00338-022-02249-4.

## Introduction

Ecosystem engineering scleractinian cold-water corals (CWCs) create biodiversity and carbon cycling hotspots in the deep and cold regions in the world’s oceans (Roberts et al. 2009; Freiwald et al. 2021; White et al. 2012). These unique ecosystems are currently under threat due to ongoing climate change (Guinotte et al. 2006; Sweetman et al. 2017). In particular, climate change leads to an increase in atmospheric carbon dioxide ($p_{{{\text{CO}}_{{2}} }}$) causing a decrease in ocean pH, known as “ocean acidification” (e.g., Doney et al. 2009; Pachauri et al. 2014). This decrease in seawater pH leads to a shoaling of the aragonite saturation horizon (ASH), thereby limiting the ability of CWCs to build their skeletons (Guinotte et al. 2006). However, the oceans are not only a sink for CO_2_, but also store atmospheric heat being increasingly produced due to greenhouse effects (Glecker et al. 2016). Recent studies have highlighted that bathyal temperatures of the North Atlantic may increase by up to 3 °C by 2100 (Sweetman et al. 2017). Thus, the combined effect of decreasing seawater pH and increasing oceanic heat content may gradually jeopardize the existence of modern CWC reefs.

The main reef-forming CWC, *Desmophyllum pertusum* (formerly described as *Lophelia pertusa*, Addamo et al. 2016), is tolerant to a broad range of bottom water temperatures (BWT, 5–15 °C, Büscher et al. 2017; Dorey et al. 2020) suggesting that, to a certain degree, CWCs have the ability to cope with thermal stress. In order to obtain environmental and oceanographic thresholds controlling the occurrence of CWCs, ecological tolerance ranges have been studied by observation of modern reef systems (Dullo et al. 2008; Flögel et al. 2014; Juva et al. 2020, 2021) or by the correlation of CWC occurrences to global datasets (Freiwald 2002; Davies et al. 2008; Davies and Guinotte 2011). Additionally, cultivation experiments have been carried out in order to define parameters that inhibit or increase calcification or growth of CWCs (e.g., Form and Riebesell 2012; Büscher et al. 2017; Gammon et al. 2018; Maier et al. 2009). Based on these findings, a variety of parameters have been identified that control the distribution of CWCs and their growth: oxygen, temperature, seawater carbonate system parameters, nutrients, salinity and seawater density (Dodds et al. 2007; Davies et al. 2008; Dullo et al. 2008; Freiwald et al. 2009; White and Dorschel 2010; Flögel et al. 2014) as well as food supply, steered by surface productivity (White et al. 2005; Duineveld et al. 2007; White and Dorschel 2010). Other studies found CWC reef growth to be controlled by the interaction between increased bottom flow and local topography (Frederiksen et al. 1992; Mienis et al. 2007; Dorschel et al. 2007; Rüggeberg et al. 2011; Juva et al. 2020; Wienberg et al. 2020). Over the last 3 million years, CWCs have been able to build mound-like structures that are also composed of hemipelagic sediments and shells of associated fauna (e.g., De Mol et al. 2002; Kano et al. 2007; Raddatz et al. 2014; Rüggeberg et al. 2007). These “mounds” are predominantly formed by *D. pertusum* with contributions of *Madrepora oculata*, *Solenosmilia variabilis*, *Batelia candina*, and *Enallopsammia profunda* (Frank et al. 2011; Muñoz et al. 2012; Hebbeln et al. 2014; Raddatz et al. 2020). Here, we refer to the classification by Wienberg and Titschack (2017) highlighting that all three-dimensional seabed structures formed by CWCs are termed mounds.

**Fig. 1:**
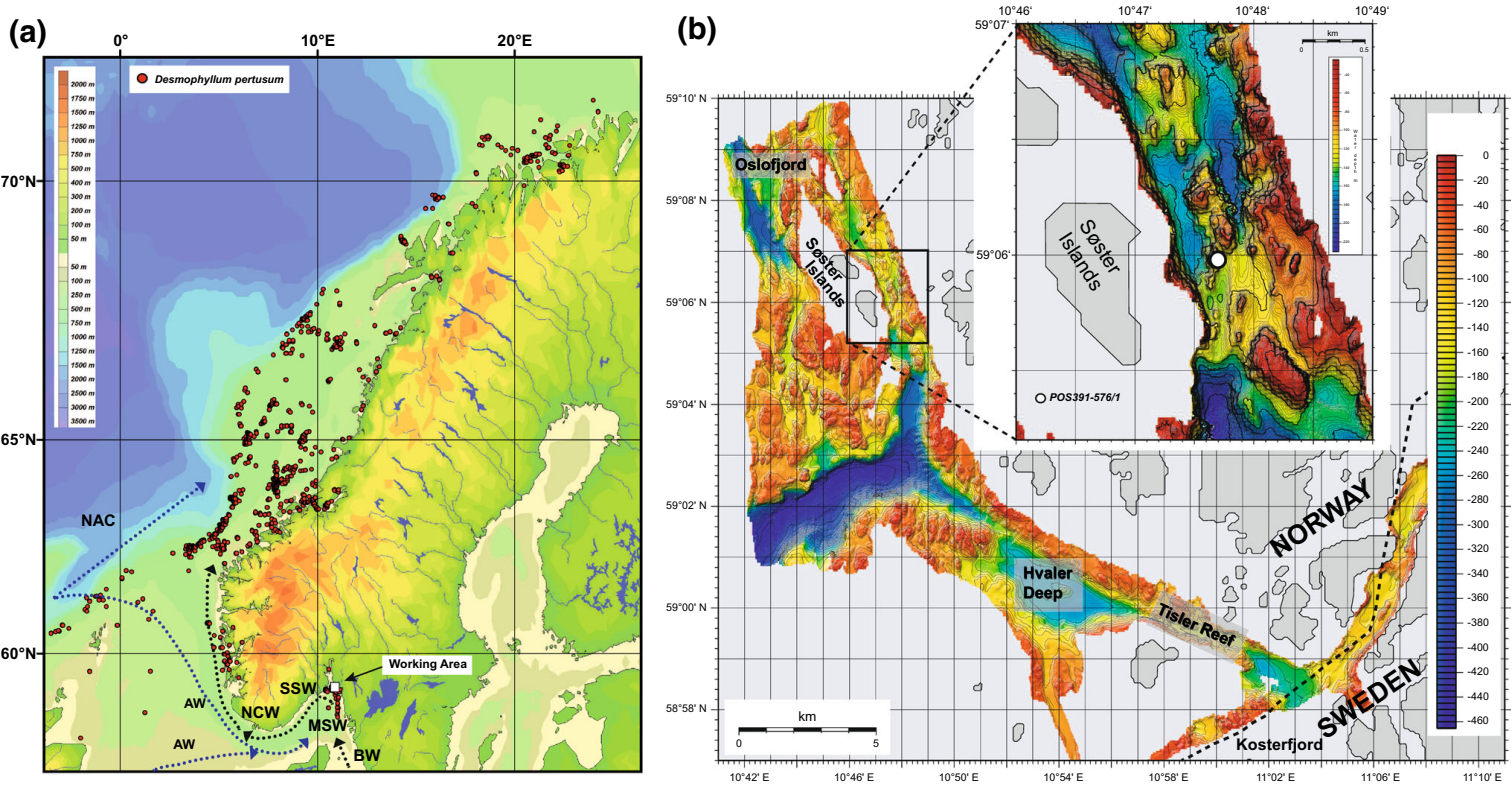
**a** Distribution of the framework-building scleractinian cold-water coral *Desmophyllum pertusum* (red dots) along the Norwegian margin (after Freiwald et al. 2021). White square is the working area in the NE Skagerrak. Also shown are surface (dotted black) and subsurface or deep currents (dotted blue). *NAC* North Atlantic Current, *AW* Atlantic Water, *BW* Baltic Water, *MSW* Mixed Surface Skagerrak Water, *SSW* Skagerrak Surface Water, *NCW* Norwegian Coastal Water. Please note for simplification, only major current important for this study are shown. **b** Detailed bathymetric map of the NE Skagerrak (**a**) and the Søster mounds east of the Søster Islands with the core location POS391-576/1. Bathymetric maps were performed during RV ALKOR cruise AL232 (Pfannkuche 2004)

On the Norwegian margin, CWC mound formation initiated after the Younger Dryas (YD) with the retreat of the Fennoscandian ice sheet at around 11 ka (Lindberg and Mienert 2005; Raddatz et al. 2016). During the Holocene their formation has been interrupted by regional climatic and oceanographic perturbations (López Correa et al. 2012; Douarin et al. 2014; Titschack et al. 2015; Raddatz et al. 2016). Modern CWC habitats off Norway extend from ~ 71° N to ~ 58° N (Fig. 1, Freiwald et al. 2021). These mounds are dominated by *D. pertusum* (Freiwald et al. 2004) and only to a minor degree by other scleractinian CWCs such as *M. oculata*. The size of these thriving mounds reaches up to 45 m in height and a lateral extent of several hundred meters (Lindberg and Mienert 2005), but often can be found in clusters with up to 35 km in length (Fosså et al. 2005). On the entire Norwegian margin CWC mounds occur in water depths mainly between 200 and 400 m (Flögel et al. 2014), but in a relatively large range of seawater salinities of 33 to 37 g/kg (Freiwald 2002) and BWTs between 4 and 8 °C (Dullo et al. 2008; Flögel et al. 2014). A very narrow seawater density (sigma theta, *σ*_Θ_) envelope of 27.5 ± 0.15 can be found at living CWC mounds along the Norwegian margin (Dullo et al. 2008; Rüggeberg et al. 2011), that triggers the accumulation of organic matter and production of sediment suspended bottom water layers (nepheloid layers, Mazzini et al. 2012), thereby fostering the flourishing states of CWCs (Flögel et al. 2014; Rüggeberg et al. 2016).

One way to reconstruct whether CWC mounds flourished in the past or not is to determine their aggradation rates (ARs, in centimetres per thousand years, cm/kyr, Frank et al. 2009; Titschack et al. 2015; Wienberg et al. 2018; Raddatz et al. 2020). During the Holocene, Norwegian mean ARs exceeded 1000 cm/kyr, close to the extension rates of individual *D. pertusum* specimens at 2.5 cm/yr (Gass and Roberts 2010). However, there is still a lack of knowledge on past temperature, density, and salinity variability in CWC mounds and such variability may have affected CWC mound formation, especially in shallow CWC habitats. Oceanographic and physicochemical properties of past water masses can be reconstructed using geochemical signatures in scleractinian CWCs (e.g., Montagna et al. 2014; Schleinkofer et al. 2019), but also in foraminifera captured in matrix sediment baffled by the coral framework (e.g., Rüggeberg et al. 2007; Margreth et al. 2009; Raddatz et al. 2011; Schönfeld et al. 2011; Stalder et al. 2014; Fentimen et al. 2020). In particular, benthic foraminiferal shells record changes in seawater temperatures in their magnesium to calcium ratios (Mg/Ca; e.g., Elderfield et al. 2010; Poggemann et al. 2018), seawater density variations in their stable oxygen isotope composition (δ^18^O; Lynch-Stieglitz et al. 1999b; Rüggeberg et al. 2016), and the combination of both proxies can be used to infer relative salinity changes (e.g., Lea et al. 2000; Nürnberg 2000). We applied these foraminifera-based proxy reconstructions to a sediment core retrieved from the shallow Søster CWC mound complex in the NE Skagerrak (Fig. 1), a CWC habitat which currently experiences large ranges in seawater temperature and salinity. The isotope and elemental proxy data are combined with computed tomography (CT) acquisitions and ^230^Th/U age determination in order to reconstruct past BWT, seawater density and salinity variability during late Holocene CWC mound formation off southern Norway.

## Cold-water coral occurrences and the hydrographic setting of the NE Skagerrak

**Fig. 2:**
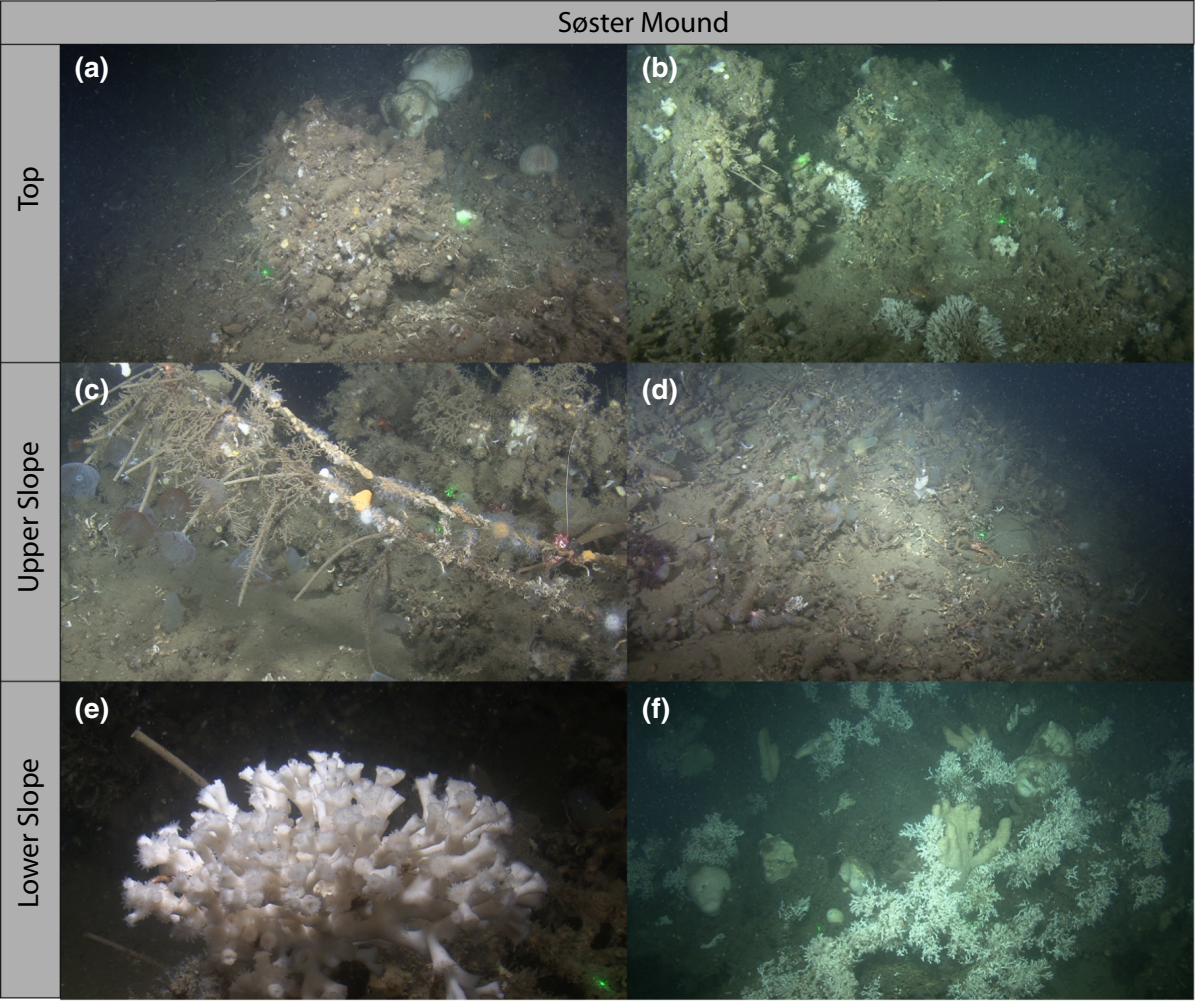
Images of *Desmophyllum pertusum* in the study area Søster mound, taken by the manned submersible JAGO. *Mound top* **a** dead *D. pertusum* framework at 88 m water depths, **b** few occurrences of living *D. pertusum*, but mostly dead coral framework plus white fan worm *Filograna implexa. Upper slope* **c** fishing line overgrown by tube worms, soft corals and bryozoans at 97 m water depth, **d** dead *D. pertusum* framework and coral rubble at 99 m water depths. *Lower slope* **e** flourishing *D. pertusum* colony extending tentacles at 107 m water depth, **f** patchy occurrences of live *D. pertusum* at 113 m water depth

CWC occurrences in the eastern Skagerrak were first reported by Wahrberg and Eliason (1926). In particular, the Oslo fjord system with elevated sills and offshore islands provides dynamic environments, but also shelter from extreme and strong currents, enabling CWC mound formation (Freiwald et al. 2004) (Fig. 1). In contrast to the open Atlantic, the Skagerrak CWC mounds occur in relatively shallow water depth between 80 and 120 m (Figs. 1, 2). The most prominent CWC habitat in this area is the Tisler Reef extending over ~ 2 km (N–S) at 90–120 m water depth. The Tisler Reef connects the Kosterfjord via the Hvaler Deep with the Oslofjord (Fig. 1). The Søster Islands south of the Oslo fjord occur on submerged drumlins (hills build by a glacier, Freiwald et al. 2004) at which the Søster CWC mound complex (east/inshore of the islands) developed, characterized by two build-ups at 88 and 110 m water depth elevating from around 140 m (Figs. 1, 2). Both summits are covered by dead corals, while the amount of living CWCs and associated fauna increase continuously further downslope (Fig. 2). Overall, the environmental and oceanographic conditions at both, the Tisler and Søster Reefs, are similar to the open Skagerrak and characterized by a strong baroclinic stratification and a relatively large temperature range (Wisshak and Rüggeberg 2006; Guihen et al. 2012). Temperature changes of ~ 7 °C over the course of 4 yr and short-term temperature shocks of 4 °C within 24 h have been recorded at the Tisler Reef, possibly exerting a strong control on coral physiology (Guihen et al. 2012).

**Fig. 3:**
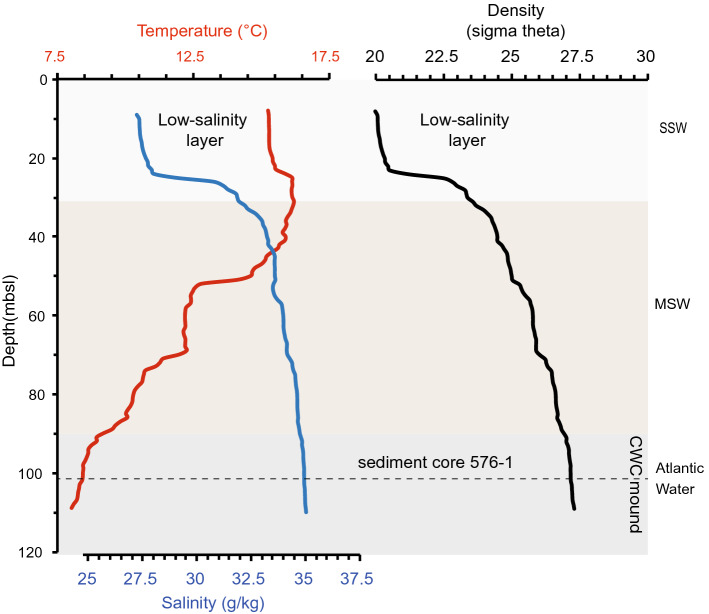
Conductivity–temperature–depth (CTD) profile taken during research cruise POS391 adjacent to sediment core 576/1. Also plotted is the corresponding seawater density profile. Shading indicate the depth ranges of the different ambient water masses according to Danielssen et al. (1997). Skagerrak Surface Water (SSW), Mixed Skagerrak Water (MSW) and the Atlantic Water. For details see text

**Fig. 4:**
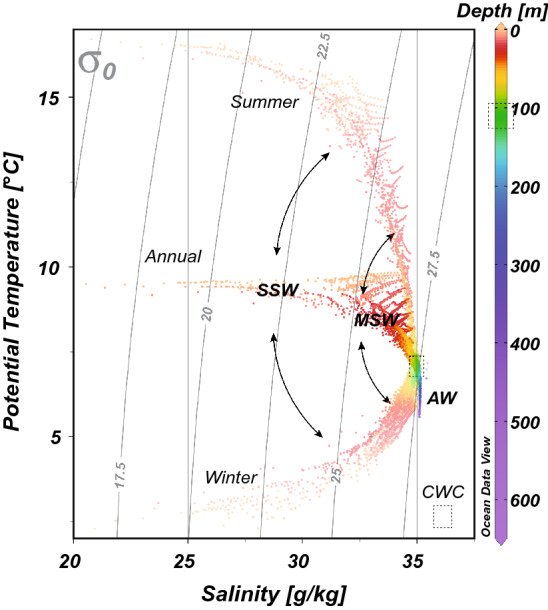
Temperature–salinity–density (TSD) plot based on annual, summer (July–September) and winter (January–March) World Ocean Atlas 2018 (0.25°) data (Boyer et al. 2018) highlighting the seasonal variability especially for the Skagerrak Surface Water (SSW) and the Mixed Skagerrak Water (MSW), while Atlantic Water (AW) stays relatively stable. Dashed box indicates the depth of CWC occurrence for the Oslofjord and Tisler Reef

The hydrography of the NE Skagerrak is mainly characterized by inflowing North Atlantic water from the North Sea overlain by brackish water from the Kattegat forming a strong haline stratification (Gustafsson 1999). The in- and outflow of North Atlantic water is intensified by a positive North Atlantic Oscillation (NAO), due to the stronger than average westerly winds, which in turn lead to higher precipitation and milder temperatures (Hurrell 1995; Hurrell et al. 2003; Winther and Johannessen 2006). Continental runoff enters the NE Skagerrak via many fjords, additionally lowering surface salinities (Winther and Johannsson 2006). Tidal currents are rather weak and residual flows are the main current strength driver along the channel axis from NE and SW dominantly controlled by either density or wind-driven sea-level height differences (Guihen et al. 2012; Lavaleye et al. 2009; Wagner et al. 2011; White et al. 2012; De Clippele et al. 2018). As a result of this the NE Skagerrak is characterized by three different water masses including the low salinity (20–32 g/kg) Skagerrak Surface Water (SSW) occupying the top ~ 30 m of the water column, the underlying Mixed Skagerrak Water (MSW) with salinities between 32 and 35 g/kg and below the saline Atlantic Water (AW > 35 g/kg, Andersson; 1996; Rohde 1996; Danielssen et al. 1997) (see Figs. 3, 4). In the transition zone of the MSW and the inflowing AW the Skagerrak CWC mounds are situated (Figs. 3, 4), but are separated from the surface by a low-salinity layer that forms an oceanographic barrier. Seasonal variations clearly affect the temperature and salinity of the upper water masses SSW and MSW, while the AW mass is more stable (Fig. 4).

## Materials and methods

During RV POSEIDON cruise POS391 in 2009, the 212-cm long sediment core 576-1 was retrieved from the fossil Søster CWC mound complex in the NE Skagerrak east of the Søster Islands (Fig. 1, Raddatz et al. 2016) from 101 m water depth (59° 11′ N, 010° 98′ E). The coring location was chosen based on visual inspection by previous dives with the manned submersible JAGO (GEOMAR) as close as possible to the observed living CWC colonies (Fig. 2). In addition, conductivity–temperature–depth (CTD) profiles have been collected using a CTD Rosette equipped with a Seabird SBE 9plus device to measure physical properties (Fig. 3).

### Computed tomography 

X-ray CT acquisitions of whole round core sections were obtained with a multi-detector Siemens Somatom Sensation 64 (Siemens, Medical Solution AG, Erlangen, Germany) installed at the University Hospital Gasthuisberg, KU Leuven (Belgium). Core sections were scanned using an X-ray source with currents of 120 kV and 135 mA. Pixel resolution of the scanned slices is 0.6 mm and slice thickness 0.9 mm with reconstruction intervals of 0.6 mm (0.628906 mm pixel size)*.* Reconstructed slices have been imported in the software Avizo (Thermo Fisher Scientific) for advanced segmentation and visualization. For both core sections, the same volume of interest has been extracted and individual slices have been filtered using a non-local means filter prior to segmentation to remove noise in the matrix sediments. Subsequently, corals were segmented from the matrix sediments using respective single thresholding followed by watershed segmentation. Prior to quantification and visualization, labeled objects smaller than 5 voxels have been removed. For each slice, the total volume % of coral fragments has been quantified (Supplementary Material, Table S2)

### ^230^Thorium/Uranium age determination 

**Fig. 5:**
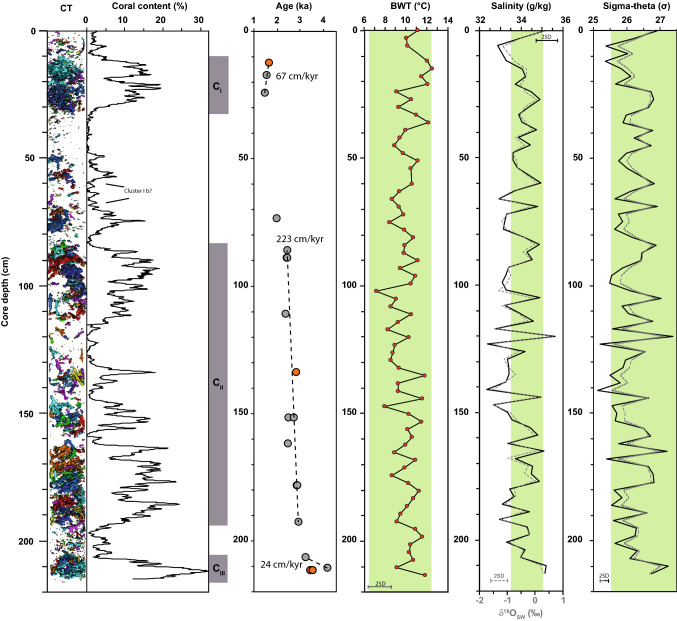
Downcore reconstruction on sediment core POS391-576/1. Computed tomography coral clast reconstructions, percentages of coral content, ^230^Th/U age determinations including ages from Raddatz et al. (2016, orange) and resulting ARs, foraminiferal Mg/Ca based bottom-water-temperature (BWT), salinity reconstruction and δ^18^O of seawater (dashed), as well seawater density reconstruction based on the method of Rüggeberg et al. (2016; solid line) and using the 4‰ fresher end member equation of Lynch-Stieglitz et al. (1999b, dashed line). The gray boxes indicate the intervals of identified coral clusters I–III. The green bars indicate the range of modern variability (e.g., Guihen et al. 2012). The plotted 2 SD uncertainties correspond to ± 1.1 °C for the Mg/Ca-based temperature reconstruction, ± 0.25 g/kg^3^ seawater density and ± 0.5 g/kg

Supplementary to the four already dated coral fragments of Raddatz et al. (2016), 15 additional ^230^Th/U age determinations were carried out in order to improve the chronology of core 576-1 (Table S1). Samples were selected according to the depositional intervals of the coral abundances based on the CT images. Prior to the analyses, all coral samples were cleaned mechanically to remove any contaminants from the skeleton surface (e.g., epibionts, borings, ferro-manganese crusts and coatings) and were then chemically cleaned including an oxidative step (50/50 mixture of 30% H_2_O_2_ and 1 M NaOH) as well as brief leach in 1% HClO_4_ (50/50 mixture with 30% H_2_O_2_) according to the protocol of Cheng et al. (2000). The measurements were carried out at GEOMAR Helmholtz Centre for Ocean Research Kiel on a multi-collector inductively coupled plasma mass spectrometer (Thermo Fisher, Neptune plus) by adapting the multi-static MIC-ICP-MS approach after Fietzke et al. (2005). Age determinations (Fig. 5; Table 1S) are based on half-lives after Cheng et al. (2013) and values for reference material HU-1 given therein (for details refer to notes presented in Table S1). The ^232^Th concentrations of all analyzed corals were always < 6 ppb, which is indicative of minor residual contamination with Th either non-carbonate phases (detritus and coating) or seawater. The initial ^234^U/^238^U activity ratio of all analyzed corals (Table S1) plot, when transferred into δ^234^U notation (i.e., ‰ deviation from secular equilibrium), within uncertainty in a narrow band of ± 10‰ compared to the value of modern seawater (145.0 ± 1.5‰, Chutcharavan et al. 2018). This finding suggests a closed system behavior for the exchange of U between the skeletons and seawater or the embedding sediment matrix. In order to verify this assumption and to estimate the reliability of the Th/U system in this setting, corals with different external preservation state from the same core depth were analyzed. This analysis resulted within uncertainty in identical U isotope signatures and ages. Hence, all ages presented in this study (Table S1) are considered reliable, especially with respect to potential variations in seawater isotope signature due to the location at the interface between Atlantic and less saline Baltic water masses.

### Aggradation rates

Coral ages from distinct clusters can also be used to calculate coral mound ARs. These clusters are stratigraphically closely related and represent periods of active mound formation. Previous studies (Frank et al. 2009; Douarin et al. 2014; Titschack et al. 2015; Wienberg et al. 2018; Raddatz et al. 2020) have calculated ARs from chronologically occurring corals within clusters thereby representing minimum and maximum ARs. Here, ARs were calculated from the maximum and minimum core depths and the corresponding ages of those cluster based on the CT images, thus representing average ARs (Wienberg et al. 2018; Raddatz et al. 2020). A prominent issue in CWC mound sediments is the possible age offset between matrix sediment and coral ages (Eisele et al. 2014; Bahr et al. 2020). However, for Norwegian CWC mounds it has been shown, by the comparison of matrix (foraminifera) and coral ages, that these are coeval to active periods of mound growth with efficient baffling of sediment by the coral framework (López Correa et al. 2012). Therefore, we assume that (matrix) sediments represent similar time periods as the coral framework.

### Geochemical proxy analyses on benthic foraminifera 

**Plate 1:**
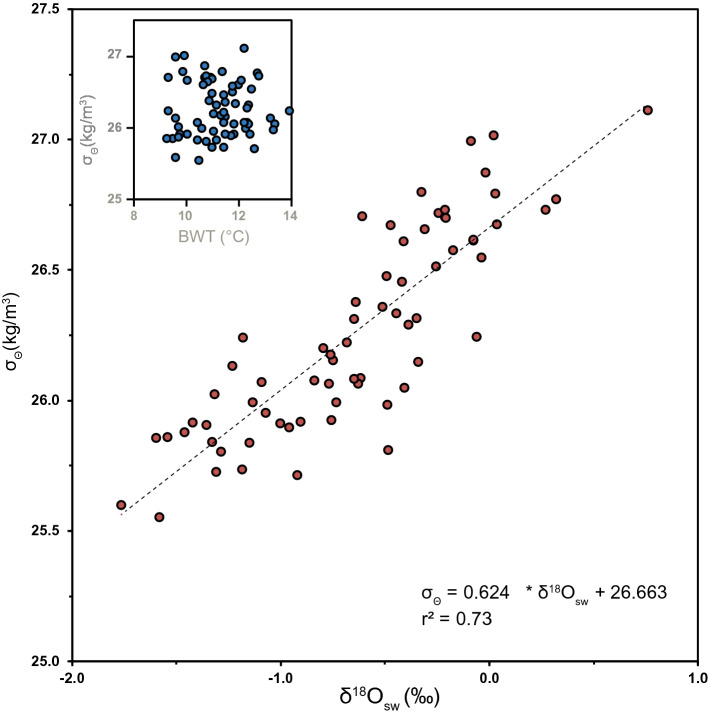
SEM pictures of the benthic foraminifera *Lobatula lobatula*. **1** Umbilical view, **2** lateral view, **3** spiral view and **4** detailed view of pyrite attachments on the foraminiferal shell

Approximately 10–15 visually well-preserved foraminiferal specimens of *Lobatula lobatula* (Plate 1) were selected from the dry sediment for analyses of stable isotope and element to calcium ratios. In order to reduce ontogenetic effects on δ^18^O_Calcite_ and Mg/Ca, we only used specimens > 315 μm (e.g., Elderfield et al. 2002). Prior to geochemical analyses, the tests were cracked carefully between glass plates and then split into two third for Mg/Ca measurements and one third for stable isotope analyses.

### Elemental ratios of benthic foraminifera and bottom water temperature reconstructions

Prior to elemental analysis, the foraminiferal tests were cleaned following established protocols including oxidative and reductive cleaning steps (Barker et al. 2003; Martin and Lea 2002). The analyses were performed on an axial viewing Varian 720 inductively-coupled-plasma-optical-emission-spectrometer (ICP-OES) at GEOMAR. The sample solution was diluted with yttrium water (concentration 112.5 μmol/l) prior to measurement in order to detect possible matrix effects during the analyses. The element/Ca measurements were drift-corrected and standardized using an internal consistency standard (ECRM 752-1, 3.761 mmol/mol Mg/Ca, Greaves et al. 2008). The external reproducibility for Mg/Ca of the ECRM standard is ± 0.1 mmol/mol (2 SD). To monitor the cleaning success of the foraminifera, Al/Ca, and Fe/Ca ratios were measured. Accordingly, we observe in the present dataset relatively high Al/Ca and Fe/Ca ratios of ~ 0.3 mmol/mol and ~ 4.0 mmol/mol, respectively (Supplementary Material, Table S3). Relatively high Fe/Ca ratios were also detected in the near-by Little Belt (IODP Exp. 347, Site M0059), and were related to the formation of (Fe) sulfides (Kotthoff et al. 2017). However, an adjusted cleaning protocol by reversing the reduction and oxidation steps of the method by Martin and Lea (2002) to remove contamination by Fe-sulfides did not result in a significant difference in Fe/Ca ratios (Kotthoff et al. 2017). In this study the increased Fe/Ca ratios appear to be the result of reoccurring pyrite on the foraminiferal shell causing the observed discrepancies from common foraminiferal Fe/Ca ratios (Barker et al. 2003; Plate 1). As pyrite does not contain considerable amounts of Mg, the Mg/Ca paleo-thermometer is not biased (see also Nürnberg et al. 2015). Furthermore, all monitored ratios such as Fe/Ca and Al/Ca do not exhibit a significant relationship to the Mg/Ca ratios (*R*^2^ < 0.4) highlighting that the Mg/Ca ratios are not influenced (Fig. S1). The Mg/Ca-BWT calibration by Quillmann et al. (2012) can be described by a linear and an exponential relationship. An ongoing discussion exists if Mg/Ca-BWT calibrations in benthic foraminifera are linear or exponential (Lear et al. 2002; Marchitto et al. 2007; Elderfield et al. 2010; Hasenfratz et al. 2017), even though exponential regressions fully explain the expected relationships by thermodynamics (Rosenthal et al. 1997). This study used the species-specific exponential calibration by Quillmann et al. (2012) for the epibenthic foraminifer *L. lobatula* (exponential fit): Mg/Ca (mmol/mol) = 1.24 ± 0.04 e^0.069 ± 0.005 * *T*^. The reproducibility (2 SD) of our Mg/Ca measurements the results in uncertainties of the reconstructed BWT of ± 1.1 °C.

### Stable oxygen isotope measurements 

The δ^18^O measurements on *L. lobatula* (δ^18^O_C_) were conducted on a Thermo Scientific MAT 253 mass spectrometer equipped with a CARBO Kiel IV device at GEOMAR. Isotope ratios were calibrated against the NBS 19 (National Bureau of Standards) and the in-house “Standard-Bremen” (Solnhofen limestone). Values are reported relative to the Pee Dee Belemnite (PDB) standard (Supplementary Material, Fig. S1; Table S3). The external reproducibility of the foraminiferal samples based on the in-house standard is ± 0.06‰ for δ^18^O (2 SD). The stable carbon isotope (δ^13^C) results will be presented elsewhere.

### Seawater density and salinity reconstructions

Regional variations of bottom water salinity were approximated from the reconstructed stable oxygen isotope ratios of the seawater (δ^18^O_SW_) (e.g., Nürnberg et al. 2021), that were calculated by combining the Mg/Ca-based temperatures and the corresponding δ^18^O_C_ values from the same foraminiferal sample. In particular, the temperature effect of the measured foraminiferal δ^18^O_C_ was removed by applying the temperature versus δ^18^O_C_ equation for cosmopolitan epibenthic foraminifera of Marchitto et al. (2014) resolved towards δ^18^O_SW_. We refrain from calculating ice-volume free δ^18^O_SW_ as the change of global ice volume over the last 4 ka appears to be negligible (Lambeck et al. 2014). The modern δ^18^O_SW_–salinity relationship (δ^18^O_SW_ = 0.26 * *S* − 8.65; *R*^2^ = 0.87; see Supplementary Material Table S5) was used to calculate absolute salinity changes from reconstructed δ^18^O_SW_ values and compared to salinity determinations based on combined Mg/Ca-temperatures and δ^18^O_C_-density reconstructions at depth of the Søster CWC mound (Tomczak 2000; based on algorithms of Fofonoff and Millard 1983).

Seawater density estimates were determined according to the established approach of Lynch-Stieglitz et al. (1999a, b) that has also been applied to CWC mounds (Rüggeberg et al. 2016; Raddatz and Rüggeberg 2021). This reconstruction is based on the fact that a change in both, seawater density and δ^18^O_C_ is controlled by salinity and temperature (Lynch-Stieglitz et al. 1999a) and that this relation is constant throughout geological time. In this study, past seawater densities were estimated following the approach of Rüggeberg et al. (2016) on the basis of a regional calibration (*σ*_Θ_ = 25.64(± 0.26) + 1.43(± 0.03) * δ^18^O_C_ + 0.21(± 0.02) * δ^18^O_C_^2^), which relies on recent δ^18^O_sw_, temperature, salinity, pressure and density data from Dullo et al. (2008), Harwood et al. (2008), Cefas CTD data of two surveys, “Cend 10/04” and “Cend 13/05” (BODC inventory) and this study (see Supplementary Material Tables S4, S5). This data set is compared to the application of Lynch-Stieglitz et al. (1999a) with 4‰ lower fresh end-member δ^18^O_C_–density relation of the global ocean (Fig. 5, *σ*_Θ_ = 25.7 + 1.0 * δ^18^O_C_ + 0.12 * δ^18^O_C_^2^). The uncertainty of the combined δ^18^O_C_ and Mg/Ca δ^18^O_SW_-reconstructions is ± 0.35‰ (see also Bahr et al. 2013; Nürnberg et al. 2015), whereas the uncertainties on the absolute salinity reconstruction are ± 0.5 g/kg following Fofonoff and Millard (1983) and the approach of Rüggeberg et al. (2016).

## Results

### CTD

The upper 25 m of the water column are characterized by a low-salinity layer with temperatures of ~ 15.3 °C and salinities as low as 27.2 g/kg (Fig. 3) which can be associated with the SSW. The seawater density also shows the low salinity layer down to a water depth of ~ 25 m, with values as low as 20 kg/m^3^. Below 25 m water depth salinity increases rapidly and reaches values of 31 g/kg. At similar depth the seawater temperature is characterized by an inversion, with temperatures increasing by up to 1 °C to maximum values of 16.3 °C. Below 40 m water depth salinity increases while temperature decreases gradually, indicating the presence of the MSW. Upper AW can be identified at ~ 100 m water depth with salinities of 35 g/kg accompanied with the presence of CWCs. The corresponding seawater density gradually increases with water depth reaching values of up 27.4 kg/m^3^. At the water depth of the sediment core 576-1 at 101 m, the temperature is 8.3 °C, the salinity 35 g/kg and the seawater density 27.4 kg/m^3^ (Fig. 3).

### Coral content, age constraints and aggradation rates

The CT based coral content reconstruction in sediment core 576-1 exhibits a large range. On average the coral content is 7%, with maximal values of 30% and minimum values of 0% (Fig. 5). The ages obtained range from 1.64 ± 0.04 ka at 12 cm to 3.40 ± 0.1 ka at 212 cm (Raddatz et al. 2016), whereas the youngest sample of 1.44 ± 0.02 ka can be found at 24 cm and the oldest samples of 4.13 ± 0.04 ka at 211 cm. Overall the coral ages exhibit three age clusters. Cluster I ranges from 12 to 24 cm with ages between 1.44 ± 0.02 and 1.64 ± 0.04 ka. Cluster II reveals ages from 2.37 ± 0.03 to 2.89 ± 0.02 ka between 89 and 193 cm, whereas Cluster III exhibits a prominent sediment disturbance or age reversal of ~ 1 kyr (3.18 ± 0.03 to 4.13 ± 0.04 ka), between 207 and 212 cm (Fig. 5). Between 17 and 193 cm core depth, the mound sequence reveals a clear continuous aggradation with on average 107 cm (including the intervals with low coral content). ARs of the three identified clusters are I = 67 cm/ka, II = 223 cm/ka and III = 24 cm/ka, resulting in an average AR of 104 cm/ka, similar to the overall sedimentation rate of 107 cm/ka (Fig. 5).

### Mg/Ca and δ^18^O ratios of benthic foraminifera

**Fig. 6:**
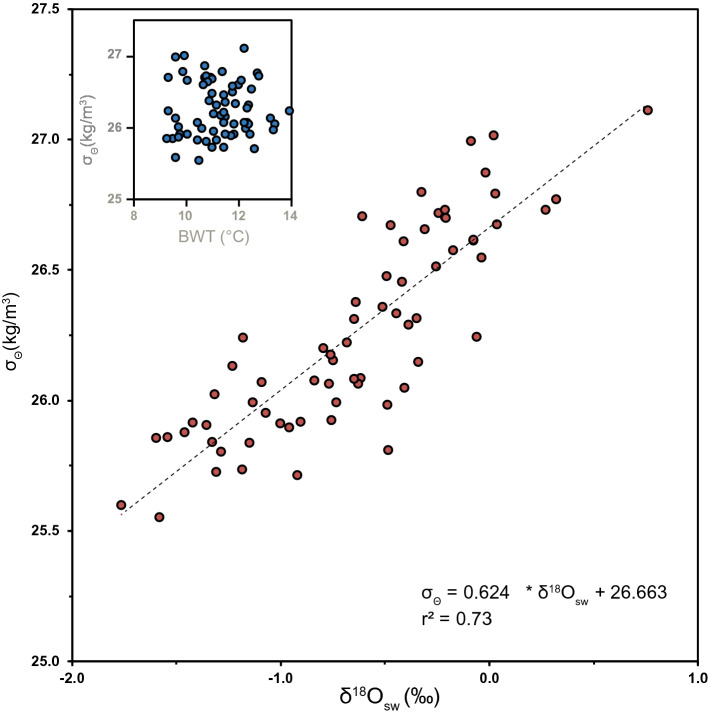
Linear relationship between δ^18^O_C_-based seawater density and δ^18^O_C_–Mg/Ca-based δ^18^O_SW_ reconstructions. Also shown is the (non) relationship between Mg/Ca-based BWT and δ^18^O_C_-based seawater density (small plot, blue; *R*^2^ = 0.73) reconstructions. The 2 SD uncertainty on the δ^18^O_SW_ is ± 0.35‰, ± 1.1 °C for the Mg/Ca-based BWT temperature and ± 0.25 g/kg^3^ for the seawater density reconstructions

Mg/Ca ratios of the benthic foraminifera *L. lobatula* reveal large variations of 0.92 mmol/mol between 3.26 and 2.35 mmol/mol. This relatively large variability of Mg/Ca ratios is prominent throughout the entire core and varies around an average value of ~ 2.7 mmol/mol. The corresponding δ^18^O_C_ ratios show a similar pattern with variations of 1.9‰ between 1.6 and − 0.3‰ resulting in an average of 0.4‰. Overall, the Mg/Ca and δ ^18^O_C_ ratios have no clear relationship suggesting that δ^18^O_C_ at ~ 100 m water depth at Søster mound is largely controlled by the δ^18^O_SW_ (salinity, respectively) and not by BWT changes (Fig. 6; Fig. S2).

### Bottom-water-temperature, seawater δ^18^O, salinity and seawater density reconstructions

The Mg/Ca–BWT relation of Quillmann et al. (2012) has been calibrated for Mg/Ca ratios of *L. lobatulus* between 1.0 and 2.4 mmol/mol (0 and 10 °C, respectively). Most of the Late Holocene Mg/Ca ratios of this study (Fig. S1) are at or outside the warmer end of the calibration line of Quillmann et al. (2012) leading to unrealistic high bottom water temperatures when using the linear calibration curve. However, their exponential fit results in reconstructed BWT with values between 7.1 and 12.5 °C and an average of 10.1 °C (Fig. 5), which is in overall agreement with instrumental data of the near-by Tisler Reef (Guihen et al. 2012).

The BWT record does not show a clear trend throughout the entire sediment core but a tendency towards higher values at the core top (Fig. 5). The δ^18^O_SW_ reconstruction reveals variations of 2.18‰ from − 1.55 to 0.63‰, with an average of − 0.6‰. Similar to the BWT reconstruction the δ^18^O_SW_ record does not exhibit clear trends but is rather characterized by the relatively large variability (Fig. 5). The seawater density reconstruction based on the foraminiferal δ^18^O_C_ also exhibits a relatively large variability of 2.3 kg/m^3^ between 25.1 and 27.4 (Fig. 5). Reconstructed salinity values differ according to the method: those reconstructed on the basis of the present-day regional S–δ^18^O_SW_ relation (δ^18^O_SW_ = 0.2547 * *S* − 8.6493; *R*^2^ = 0.87; see Supplementary Data 2) vary between 27.87 and 36.45 g/kg (*Δ* = 8.57 g/kg) and have a large uncertainty of ± 3.2 g/kg, while the ones reconstructed on the basis of Mg/Ca, seawater density and water depth (pressure) following Fofonoff and Millard (1983) show a smaller variability of 3.28 g/kg ranging between 32.25 and 35.63 g/kg with an accuracy of ± 0.5 g/kg (Fig. 5, Supplementary Material). Interestingly, similar variations have been also measured today in the nearby Tisler-Reef (e.g., BWT = 6.44–12.39 °C, salinity = 33.57–35.10 g/kg; seawater density = 25.46–27.41 kg/m^3^; Guihen and White 2012), highlighting the reliability of the foraminiferal-based reconstructions.

## Discussion

### Søster mound development during the Late Holocene

Accumulation rates identify periods of active coral mound formation (Titschack et al. 2015; Wienberg et al. 2018; Raddatz et al. 2020), whereas appraisals of the percent composition of corals gives insights into the importance of corals as mound builders (Foubert and Henriet 2009; Van der Land et al. 2011; Douarin et al. 2013; Titschack et al. 2015; Raddatz et al. 2020). In comparison to other CWC mounds the average ARs of Søster mound (Fig. 5) are higher (on average 104 cm/kyr) than those determined for the Rost Reef (off Norway, ~ 52 cm/kyr, Titschack et al. 2015), the Rockall Bank (NE Atlantic, 37 cm/kyr, Frank et al. 2009), and offshore Brazil (30 cm/kyr, *S. variabailis*, Raddatz et al. 2020), but rather similar to those observed in the Porcupine Seabight (83 cm/kyr, Frank et al. 2009) and Urania Bank/Mediterranean Sea (111 cm/kyr, Titschack et al. 2016). However, the AR found here are significantly lower than those determined in glacial CWC mounds off Mauretania and Holocene CWC mounds off northern Norway (Traenadjupet, Stjernsund) with ARs of 444 cm/kyr (Wienberg et al. 2018; Titschack et al. 2015). High ARs can be attributed to favorable environmental conditions and may represent the growth rates of individual scleractinian CWCs (Titschack et al. 2015; Raddatz et al. 2020). In particular, off Norway and Mauretania CWC mound ARs are on a similar order of magnitude as growth rate estimates of CWCs and therefore, appear to represent the upper limit of CWC mound aggradation, assuming that CWC mound growth never outpaces CWC growth (Titschack et al. 2015). A lower AR of the Søster CWC mound relative to other Holocene Norwegian CWC mounds implies that environmental conditions were not optimal for rapid mound aggregation. Differences in ARs of Clusters I, II and II sequence may thus be attributed to changes in environmental conditions. Cluster II is characterized by the highest ARs, but shows variations in coral quantities of similar magnitude as for the entire mound sequence (Fig. 5). This suggest that even within one period of enhanced formation environmental conditions were not optimal.

In general, CWC habitats are characterized by a strong current regime (Davies et al. 2009a, b; Rüggeberg et al. 2005; Mienis et al. 2007) that maintain large amounts of particles in suspension. Coral mound constructors benefit from an increased sediment supply (Wheeler et al. 2008), whereas reduced current intensities accompanied with high sedimentation may bury the corals (Dorschel et al. 2005; Rüggeberg et al. 2007; Mienis et al. 2007). Therefore, periods of enhanced sediment accumulation with only limited amounts of corals may be related to protracted periods of reduced current strength that favored the settlement of fine-grained material and/or periods of enhanced sedimentation (runoff/erosion).

Changes in sedimentation pattern may also partly be associated with changes in water depth and hence with the postglacial isostatic uplift of the Fennoscandian shield. The shoreline tilt gradient indicates that most of this uplift occurred prior to 4 ka bp and uplift occurred linearly in the NE Skagerrak after 4 ka bp with a rate of ~ 5 mm/yr (Lindberg et al. 2007; Rosentau et al. 2012). Assuming this uplift rate to be constant for the last 3.5 kyr the Søster mound would have initiated in ~ 17.5 m deeper water depth and thus would have been bathed directly in the strong current flow of the North Atlantic water (Figs. 3, 4) suggesting a strong water mass control on CWC mound formation.

### Environmental control of Søster mound

#### Temperature control

The distribution of CWCs and their reefs/mounds has been shown by predictive habitat modelling to be controlled by seawater temperature and salinity (Davies et al. 2008; Davies and Guinotte 2011). Modern CWC habitats off Norway in the aphotic Zone are accompanied by a strong hydrodynamic regime and experience relatively large variations in seawater temperature (Godø et al. 2012; Van Engeland et al. 2019; Juva et al 2020; Rüggeberg et al. 2011). For example, at the Hola Reef seasonal temperature variations of 4.5 °C (5.5–9.0 °C) were recorded and even 4 °C at Tisler Reef within 24 h (Guihen et al. 2012). How these temperature variations impact coral physiology and reef growth is still poorly constrained. Short-term experiments only found increased mortality rates at temperatures > 14 °C (Brooke et al. 2013; Lunden et al. 2014), whereas long-term experiments did not observe a change in respiration rate at similar temperatures (6–12 °C) as observed in a study over a period of three months (Naumann et al. 2014). A recent on-board experiment with *D. pertusum* did not find an increase in mortality with increasing temperatures from 5 to 15 °C (Dorey et al. 2020). Past BWT variability at Søster mound of < 6 °C are similar in all clusters (Fig. 5). As such a variability is within the known range of *D. pertusum* occurrences, we suggest that temperature is not a primary controlling parameter for CWC mound formation. Furthermore, our BWT reconstruction supports the newly performed temperature experiments by Dorey et al. (2020) implying that CWCs off Norway are acclimatized and/or have the ability to acclimatize to large temperature variations.

#### Seawater density and salinity changes

Throughout the entire mound sequence, salinity inferred from δ^18^O_SW_ exhibit large variations, suggesting that salinity variability exerts a strong control on the water mass stratification over the CWC habitats. Seawater salinity has been shown to be one of the main parameters for the prediction of suitable CWC habitats (Davies and Guinotte 2011), where different species of scleractinian CWCs prefer a limited range of seawater salinity from 34 to 37 g/kg. However, in situ observations at different flourishing *D. pertusum* reefs exhibit relatively large ranges from 31.7 to 38.8 g/kg (Freiwald et al. 2004) implying that salinity is not a primary control on the growth of CWC reefs (Dullo et al. 2008). Estimating the overall relative seawater salinity variability at Søster mound by using the calculated 2.5% range in δ^18^O_SW_ results in ~ 5 g/kg (Fig. 6, Schmidt et al. 1999), which is smaller than the tolerated range of modern CWCs and CWC reefs (Dullo et al. 2008; Freiwald et al. 2004).

Nevertheless, our dataset demonstrates that salinity exerts a strong control on the density of seawater in the region (Fig. 6). In the NE Skagerrak bottom-waters are dominated either by a warm saline North Atlantic source or a cold and fresh Baltic source, even though short-term, rapid events with warm and less saline waters have been documented at the close-by Tisler Reef (Guihen et al. 2012). Between 4 ka and 1.5 ka these different water mass sources likely resulted in the observed variations in seawater density of up to 2.2 kg/m^3^ at Søster mound (Figs. 5, 6). Today the inflow of dense AW is accompanied by an increase in current strength and a supply of fresh oxygen beneficial for the corals (Guihen et al. 2018). Furthermore, the North Sea and the Northeast Atlantic are the only sources of coral larvae migrating into the NE Skagerrak (Dahl 2013; Fox et al. 2016). These regions are atmospherically and oceanographically strongly connected via the NAO (Winther and Johannessen 2006; Fox et al. 2019). Therefore, periodically (NAO-driven) inflow of larvae from the North Atlantic CWC provinces may have supported continuous CWC growth throughout the Holocene (Mikkelsen et al. 1982; Raddatz et al. 2016). In contrast, periods of lower seawater density are accompanied by a decrease current strength resulting in a reduced replenishment of oxygen being strongly unfavorable for CWCs. During periods of reduced current flow only little water mass movement across the coral reef results into an increased descent of zooplankton mass and organic matter (Diesing et al. 2021; Guihen et al. 2018). Scleractinian CWC have been shown to consume zooplankton (e.g., *Artemia salina*, Naumann et al. 2011), which may suggest that the increased zooplankton load during times of reduced current strength may counteract (at least partly) unfavorable conditions.

However, the supply of organic matter and food may instead be associated with periods with a higher seawater density and the build-up of a water mass boundary. Generally, at flourishing CWC habitats seawater density is characterized by a steep gradient within a short bathymetric range (Dullo et al. 2008; Hebbeln et al. 2014). Large CWC occurrences associated with a strong density gradient have also been reported from the Mediterranean Sea (Freiwald et al. 2009) and the Gulf of Mexico (Davies et al. 2010; Hebbeln et al. 2014), although the absolute sigma-theta values differ (higher and lower) from those initially reported by Dullo et al. (2008). Nevertheless, paleoceanographic reconstructions using oxygen isotopes of benthic foraminifera from CWC mounds on the Irish margin reveal that mound formation always occurred within a very narrow seawater density envelope of *σ*_Θ_ between 27.3 and 27.7 kg/m^3^ (Rüggeberg et al. 2016; Raddatz and Rüggeberg 2021). Such narrow envelopes in seawater density highlight that flourishing CWC reefs and fast mound build-up appear preferentially near water mass boundaries characterized by a strong density gradient, where organic matter is carried along bottom and intermediate nepheloid layers and where internal waves propagate and reflect (e.g., White et al. 2007; Dullo et al. 2008; De Mol et al. 2011; van der Kaaden et al. 2020; White and Dorschel 2010). The ARs of NE Atlantic CWC mounds are smaller (15 cm/kyr, Titschack et al. 2009) than those observed at Søster mound. However, at the shallow Søster mound environmental conditions and especially sediment supply are not comparable to those of the CWC mounds at > 800–1000 m water depth in the North Atlantic. Nevertheless, we suggest that the interplay between inflowing Atlantic and the outflowing low saline Baltic water generated short-periods of relatively stable water mass boundary conditions between the MSW and the AW at ~ 100 m water depth (Figs. 3, 4), which in turn favored the accumulation of organic matter and thus CWC growth and CWC mound formation.

## Conclusion

Our results reveal that the Søster mounds have been influenced by relatively large temperature, salinity and seawater density variations between 4 ka and 1.5 ka. In this respect, bottom water temperature is not the prime controlling factor for mound formation. Instead, pronounced seawater density variations induced by high amplitude salinity variations likely caused by the interplay between the Atlantic inflow and cold-fresh Baltic seawater have been the primary control on CWCs in the region. The Atlantic Inflow is characterized by a high current flow leading to an enhanced supply of oxygen and also carries larvae from other Atlantic CWC provinces into the NE Skagerrak and have formed a water mass stratification beneficial for the food supply for CWCs. Whereas a stronger influence of fresh Baltic Water may have hampered CWC growth.

## Supplementary Information

Below is the link to the electronic supplementary material.

Supplementary file1 (EPS 852 kb)

Supplementary file2 (EPS 729 kb)

Supplementary file3 (XLSX 367 kb)

Supplementary file4 (XLSX 15 kb)

Supplementary file5 (XLSX 12 kb)

Supplementary file6 (XLSX 29 kb)

Supplementary file7 (XLSX 28 kb)

## Data Availability

The datasets as well as additional figures for this study can be found in the Supplementary Material associated to this article and will be made available at www.pangaea.de.
